# Executive Function Improvement for Children with Autism Spectrum Disorder: A Comparative Study between Virtual Training and Physical Exercise Methods

**DOI:** 10.3390/children9040507

**Published:** 2022-04-03

**Authors:** Chaoxin Ji, Jun Yang, Lin Lin, Song Chen

**Affiliations:** 1 Department of PE, Northeastern University, Shenyang 110819, China; chensong@pe.neu.edu.cn; 2 College of Information Science and Engineering, Northeastern University, Shenyang 110819, China; yangjun@mail.neu.edu.cn; 3 School of Social and Political Science, University of Glasgow, Glasgow G12 8QQ, UK; 2514029L@student.gla.ac.uk

**Keywords:** children with ASD, executive function, virtual training, physical exercise

## Abstract

This study evaluated and compared the effects of virtual training and physical exercise on the executive function of children with autism spectrum disorder (ASD). After screening, the final analysis of this study was conducted on three groups: a virtual training group (*n* = 34), a physical exercise group (*n* = 33), and a control group (*n* = 33). The experiment was conducted for nine weeks, of which the virtual training group and physical exercise group were conducted three times a week for one hour each time during the first six weeks, while the control group did not conduct virtual training nor physical exercise. During the last three weeks (week 6 to week 9), virtual training and physical exercise were not performed on all three groups. The three main components of executive function (working memory, inhibition, flexibility) of children with ASD were measured before the intervention, after the intervention (week 1 to week 6) and in the last three weeks (week 6 to week 9). The final results are that firstly, the executive function of the virtual training and physical exercise groups were simultaneously improved after the intervention. Secondly, after the intervention stopped, the executive function of the virtual training and physical exercise groups showed a downward trend. Therefore, the study concludes that the application of virtual training and physical exercise can effectively enhance the executive function of children with ASD.

## 1. Introduction

The symptoms of autistic children mainly include language disorder, social disorder and obsessive-compulsive-based repetitive behaviors. At present, the common view for the cause of autism is neurodevelopmental defect. Increasing evidence now shows that the executive function of children with autism spectrum disorder (ASD) has certain gaps compared with the children of typical development [1]. Executive function, by definition (Handbook of Clinical Neurology Handbook of Clinical Neurology), is a high-level cognitive process that enables people to complete complex cognitive processes. With three core functions, namely working memory, inhibition and flexibility, it has a regulating effect on people’s behavior with a mediating role in sensory processing and behavioral performance [2]. Decreased executive function can lead to cognitive and emotional disorders [3]. Notably, children’s executive function is malleable. The plasticity of executive function can be altered by external stimuli (such as exercise) and age [4,5]. In terms of the external stimuli, if the training is conducted on computer, it can effectively improve the neural response and repetitive behavior of children with ASD [6]. However, although the current research is adequate, there is still insufficiency in this regard. For instance, Hill reviewed cognitive behavioral studies among planning, mental flexibility and inhibition in ASD. He pointed to the need for more detailed research protocols to study the relationship between ASD and executive function [7]. Further study had pointed out that the executive function of ASD patients may be different from the executive function of individuals who went through typical development [8]. In a comparative study of executive function between ASD patients and patients who grew typically, Gisbert’s research [9] showed that the executive function of ASD patients was not related to psychiatric symptoms, and that the executive function of ASD patients was not different from the executive function of individuals with typical development. Through virtual driving training, Patrick [10] found that the executive function of adolescents with ASD was no different from that of adolescents with typical developmental characteristics. This discrepancy may be due to inconsistent measures of executive function. Therefore, the study of executive function in children with ASD needs to be perfected by rethinking measurement standard. 

Research in recent years has shown that physical exercise as a means with almost no side effects can intervene in children with ASD. For instance, studies have shown that mini-basketball and bicycle training can significantly improve the executive function of children with ASD [11,12]. Macoun [13] used game-based cognitive training to train children with ASD and found that it can effectively improve the executive function of children with ASD, and it also has a significant migration effect. However, there are research contradictions. Firstly, physical exercise intervention effects on the working memory of executive function are contradictory. For example, a meta-analysis showed that physical exercise can effectively improve the inhibition and working memory of children with ASD [14]. However, another meta-analysis showed that while physical exercise can significantly improve the inhibition and flexibility of children with ASD, there was no improvement in working memory [15]. Secondly, there were also contradictions about whether virtual training can improve the execution function. For example, McCord [16] applied virtual training to train the elderly, and found that after virtual training, the executive function of the elderly was effectively improved, but after stopping training for one month, the working memory of the elderly regressed to the baseline level. Another study showed that virtual training has a certain effect on improving the executive function of elderly people over 50 years old [17]. Contrary to his research, Mayer [18] found that the existing virtual training does not improve the executive function of adolescents. In addition, Parong’s research [19] demonstrated that different virtual training may have different effects on executive function. Although most studies have proved the effect of physical exercise on the improvement of executive function of children with ASD, the above studies show that the research on the effect of virtual training on executive function has not yet formed a unified opinion, so in-depth research is needed to solve these academic disputes. At present, with the development of electronic information technology, researchers are using virtual training to intervene with children’s executive function. However, the research on the intervention of virtual training in the executive function of children with ASD was relatively rare, especially the lack of research on the horizontal comparison between virtual training or physical exercise. Moreover, the current research on the comparative study of pre-test and post-test detection of executive function was significantly little; only a few propose re-measurement of skills in children with ASD after the training stopped [20]. Therefore, it is necessary to conduct high-quality comparative research to compare the effects of virtual training and physical exercise respective effects on the training effects of children with ASD. This study introduces virtual football games as a virtual training method. 

In order to analyze the effect of virtual training and physical exercise respective effects on the executive function of children with ASD, we intervened in the form of virtual training and physical exercise on children with ASD to observe the effect of improvement on the executive function of children with ASD. Therefore, for the test of this study, we adopted repeated measurements to observe the improvement of the executive function of children with ASD, which were measured once before the start of the training; once at the end (at six weeks); and three weeks after the end of the experiment. Our study hypothesized that both physical exercise and virtual training would improve executive function in children with ASD. When training was stopped, executive function in children with ASD decreased, but not to baseline levels. Based on this, we designed an experiment on the intervention effect of both physical exercise and virtual training on children with ASD.

## 2. Materials and Methods

### 2.1. Research Design

This study adopted the research method of a randomized controlled experiment. According to the number of selected children and gender, they were randomly assigned into three groups. The principle of grouping is that the gender ratios of the three groups were as equal as possible. The specific information of the three groups is shown in Section 2.2 and Section 2.3.

### 2.2. Participants

A priori power analysis (G*Power 3.1.9.7) was used to calculate the study’s required sample size. The parameters we chose were: (1) Effect size f = 0.25; (2) α err prob of 0.05; (3) power of 0.85; (4) number of groups of 3; (5) number of measures of 3. After calculation, 63 sample sizes can produce statistical significance. Considering that children may withdraw from the experiment for some reasons while participating in the experiment, it was necessary to expand the sample size appropriately. We expected an attrition rate of around 40% per group, so a minimum sample size of 88 was required.

**Figure 1:**
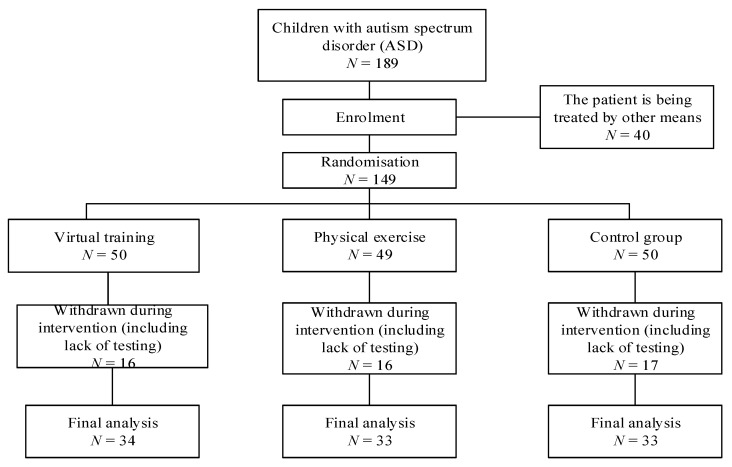
Participant flowchart across the study.

According to the needs of the research, we visited the Orphan School. In this school, the prevalence of children with ASD is high. All subjects met the current ICD-10 criteria for clinical diagnosis of “pervasive developmental disorders (PDD)” according to the World Health Organization’s (WHO) International Classification of Diseases, 10th edition (ICD-10). For children with ASD, those who met the following conditions were excluded: (1) have obvious intellectual disability; (2) have participated in or regularly take virtual training; (3) regularly exercise more than two times a week; (4) physical conditions that meant were not allowed to participate in sports activities (such as heart disease, fractures, etc.); (5) suffering from a variety of mental system diseases. We finally analyzed 100 children with ASD (58.7% more than the required overall sample size), among which the virtual training group had 34 (m = 20, f = 14), the physical exercise group had 33 (m = 17, f = 16), and the control group had 33 (m = 18, f = 15) (Figure 1). Before training, this research required their guardians to sign the proof of informed consent, with the consideration of the age of the ASD children. The information regarding age of subjects was recorded. Age, weight, height and body mass index (BMI) were measured for each child (Table 1). The Childhood Autism Rating Scale [21] (CARS) was used to evaluate children’s behavior. The scale consists of 15 items, each of which had a score of 1–4, and the total score was the sum of the items scores. If the score was greater than 30, it can be considered as ASD. A score between 30 and 36 was considered as mild to moderate ASD, and a score greater than 36 was considered as severe ASD.

### 2.3. Participant Characteristics

Results have shown that there were no significant differences in age (F(2,97) = 0.333, *p* > 0.05), gender (χ^2^(2) = 0.366, *p* > 0.05), height (F(2,97) = 1.312, *p* > 0.05), weight (F(2,97) = 1.205, *p* > 0.05), BMI (F(2,97) = 0.977, *p* > 0.05) or CARS (F(2,97) = 0.903, *p* > 0.05) in the different groups, indicating that the three groups have good homogeneity. The relevant results are shown in Table 1.

### 2.4. Study Methodology

For the game selection process, in order to be able to meet the needs of children, we chose the Xbox360 football game, which provided virtual training to practice reaction speed. Children needed to use various tactics and techniques in the game to hit the ball into the opponent’s goal. The game was divided into three levels, and we choose the lowest level of difficulty to play. Physical exercise group selected football as the training content, and mainly used football training methods, such as passing exercises, shooting exercises, and coordination exercises. The intervention group did not undergo virtual training and football training, only arranged psychological counseling and treatment. The experiments in all groups were completed under the supervision of a physical education teacher. The virtual training group and physical exercise group trained three times a week, one hour training each time, for six weeks in total. The exercise intensity of the physical exercise group was monitored with the Brog RPE scale [22]. After monitoring, it was found that the Brog RPE value of the sports intervention group was 10–13, which was basically in the range of moderate aerobic exercise intensity. The virtual training group was a handle game, and only needed to sit in the position to play the game, thus its exercise intensity was low. For all three groups of children with ASD, they all received psychological counseling organized by a unified organization. The contents of psychological counseling in each group were the same, about half an hour each time. The study has been approved by the Ethics Committee of Northeastern University. The study procedure was in accordance with the ethical standards of the institutional and national research committee, and with the 1964 Helsinki declaration and its later amendments or comparable ethical standards.

### 2.5. Production and Presentation of Executive Function Test Materials

All tests were conducted in classrooms, and children with ASD received executive function tests before training, at the sixth and ninth weeks. Each task took about 5 min. All test tasks were carried out in sequence according to the procedure.

In terms of the working memory test, it was tested by memorizing digital memory tasks backwards [23]. This test has been applied to the test of executive function of autism and has been recognized [24]. In the test, numbers were rendered at a rate of 1 per second, with an initial length of 2 and a maximum length of 9. After the presentation was completed, autistic children need to memorize numbers backwards. If the participant made a mistake in the sequence of two numbers with the same length, the test stopped and the length of the longest correct repeat sequence was recorded. The longer the sequence length, the better the working memory. The scoring rule was that one point was awarded for each correct record of a number string, and the score was between 0–16 points.

**Figure 2:**
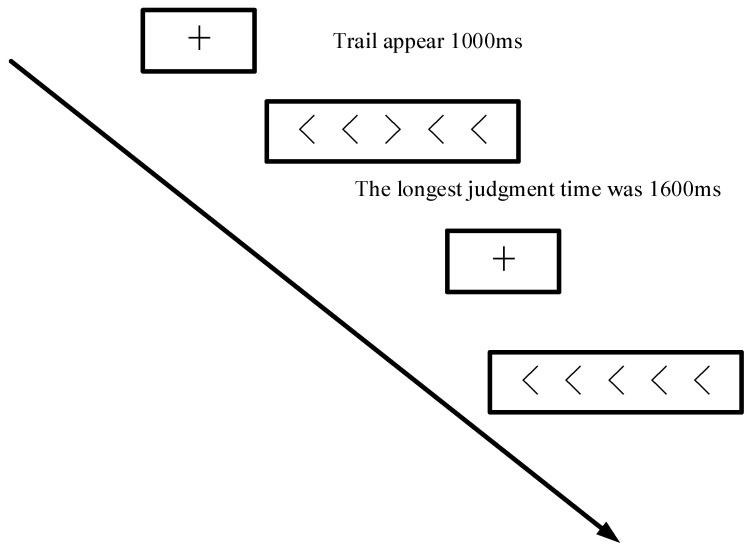
Inhibition test flowchart.

In terms of the inhibition test, it was an adapted side inhibition task flanker test [25]. The adapted flanker task proved to be more suitable for local domain usage [26,27]. Using E-prime 2.0 to present the test material, there was five symbols in the middle of the screen (such as < < < < <, >> < >>), and the time for each trail to appear was 1000 ms (Figure 2). The children with ASD were to judge the middle symbol for the direction, and whether the opening was to the left or right. The longest time for judgment was 1600 ms. If no judgment was made within 1600 ms, it was regarded as a judgment error. The calculation method was the accuracy of the symbol. There were 10 trails in the exercise section, with feedback (correct or wrong). There was a total of 60 trails in the formal experiment part. After the subjects completed 30 tests, they had a rest period of 2 min. After the rest, they performed the remainder of the test.

In terms of the flexibility test, it used the classic version of the Stroop color-word test (SCWT) for flexibility testing [28]. The SCWT has been proved to be able to be used in the test of children with ASD [29,30]. In this test, we used the verified Chinese version of SCWT [28]. The children read three different tables. There were two conditions for the three tables, which were consistent and inconsistent. There were two tables under the same condition. The first table writes the name of the color in black font; the second table has the color corresponding to the first table. The third table was inconsistent form (such as written the word ‘red’ in green ink). In the test, the subjects were asked to say the color of the words as soon as possible, regardless of the words. In this test, we only count the answer error rate to reflect the flexibility of children with ASD.

### 2.6. Statistical Analysis

Statistical analysis of data was carried out by SPSS26.0. First, all means and standard deviations were statistically analyzed using standardized statistical methods. The normal distribution of the data used the Shapiro–Wilk test, and the homomorphic distribution used the Levene test. The executive function test used 3 group (virtual training group/physical exercise group/control group) × 3 time (pre/6 weeks/9 weeks) × 3 core function (working memory/inhibition/flexibility) mixed analysis of variance. Among them, time was a factor within the group, and training was a factor between groups. Mauchly’s test of sphericity was used, together with the non-conforming sphericity test, and then Greenhouse–Geisser was used for analysis. Bonferroni post-mortem analysis was used, where significant differences occurred. Partial eta squared ($\;\eta_{p}^{2}$) calculated the effect size of significant main effects and interactions.

## 3. Results

### 3.1. The Impact of Different Training Methods on Executive Function of Children with ASD

Before training, the variance analysis was performed on each function of the pre-tested executive function of each experiment, and it was found that pre-test scores of the working memory, the inhibition, and the flexibility were not significantly different (F1(2,97) = 0.864, *p* = 0.425; F2(2,97) = 2.075, *p* = 0.131; F3(2,97) = 0.336, *p* = 0.715), which showed that there was no difference in the level of executive function of children in each group before training. In order to explore the influence of different training methods on the executive function of children with ASD, a mixed variance design of three groups (virtual training group/physical exercise group/control group) × three times (pre/6 weeks/9 weeks) was adopted, and training was used as the inter-subject variable. Time was used as the internal variable of the subject, and the dependent variable was each function of the executive function.

#### 3.1.1. The Impact of Different Training Methods on the Working Memory of Children with ASD

**Figure 3:**
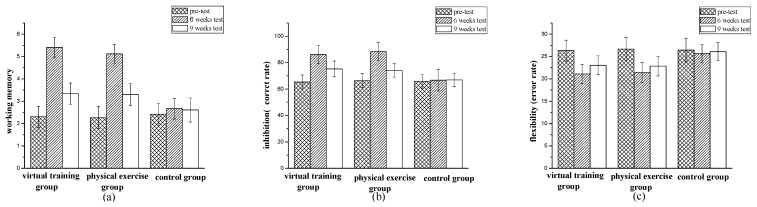
Three core functions of executive function: (**a**) working memory; (**b**) inhibition (correct rate); (**c**) flexibility (error rate).

In order to investigate the effect of different groups training on working memory of children with ASD, the mixed variance analysis found that the main effect of measuring time of working memory was significant, F(2,96) = 404.88, *p* < 0.001, $\;\eta_{p}^{2}=0.894$. After a simple effect analysis, it was found that the working memory of the second measurement (M = 4.34, SD = 1.32) was significantly better than the working memory of the third measurement (M = 3.03, SD = 0.60), and both were significantly better than the previous measurement (M = 2.34, SD = 0.47). The interaction effect between measurement time and different training methods was significant, F(4,194) = 30.57, *p* < 0.001, $\eta_{p}^{2}=0.387$. With post-hoc simple effect analysis, it was found that there was no significant difference in working memory between the virtual training group and the physical exercise group (*p* = 0.878). There was significant difference between the virtual training group and the control group (*p* < 0.001). There was significant difference between the physical exercise group and the control group (*p* < 0.001). It showed that both virtual training and physical exercise can effectively improve the working memory of children with ASD, and there was no significant difference. Intra-group comparison: the second measurement of the virtual training group and physical exercise was better than the third measurement. Both were better than the previous measurement, and there was a significant difference (*p* < 0.05). There was no significant difference between the three measurements in the control group (*p* > 0.05) (Figure 3a).

#### 3.1.2. The Impact of Different Training Methods on the Inhibition of Children with ASD

In order to investigate the effect of different group training on the inhibition of children with ASD, the mixed variance analysis found that the main effect of measuring time of inhibition was significant, F(2,96) = 1913.81, *p* < 0.001,$\;\eta_{p}^{2}=0.976$. After a simple effect analysis, it is found that the inhibition of the second measurement (M = 79.43, SD = 8.60) was significantly better than the inhibition of the third measurement (M = 71.55, SD = 4.90), and both were significantly better than the previous measurement (M = 65.80, SD = 4.87). The interaction effect between measurement time and different training methods was significant, F(4,194) = 58.16, *p* < 0.001, $\eta_{p}^{2}=0.545$. With post-hoc simple effect analysis, it was found that there was no significant difference in inhibition between the virtual training group and the physical exercise group (*p* = 0.287). There was significant difference between the virtual training group and the control group (*p* < 0.001). There was significant difference between the physical exercise group and the control group (*p* < 0.001). It showed that both virtual training and physical exercise can effectively improve the inhibition of children with ASD, and there was no significant difference. Regarding intra-group comparison, the second measurement of the virtual training group and physical exercise was better than the third measurement. Both are better than the previous measurement, and there was a significant difference (*p* < 0.05). There was no significant difference between the three measurements of the control group (*p* > 0.05) (Figure 3b).

#### 3.1.3. The Impact of Different Training Methods on the Flexibility of Children with ASD

In order to investigate the effect of different group training on flexibility of children with ASD, the mixed variance analysis found that the main effect of measuring time of flexibility was significant, F(2,96) = 187.65, *p* < 0.001, $\;\eta_{p}^{2}=0.796$. After a simple effect analysis, it was found that the flexibility of the second measurement (M = 22.62, SD = 2.16) was significantly better than the flexibility of the third measurement (M = 24.08, SD = 2.05), and both were significantly better than the previous measurement (M = 26.47, SD = 1.60). The interaction effect between measurement time and different training methods was significant, F(4,194) = 17.93, *p* < 0.001, $\eta_{p}^{2}=0.270$. Using post-hoc simple effect analysis, it was found that there was no significant difference in flexibility between the virtual training group and the physical exercise group (*p* = 0.457). There was a significant difference between the virtual training group and the control group (*p* < 0.001). There was significant difference between the physical exercise group and the control group (*p* < 0.001). It showed that both virtual training and physical exercise can effectively improve the flexibility of children with ASD, and there was no significant difference. Regarding intra-group comparison, the second measurement of the virtual training group and physical exercise group was better than the third measurement. Both are better than the previous measurement, and there was a significant difference (*p* < 0.05). There was no significant difference between the three measurements in the control group (*p* > 0.05) (Figure 3c).

#### 3.1.4. Analysis on the Improvement of Executive Function of Children with ASD

**Figure 4:**
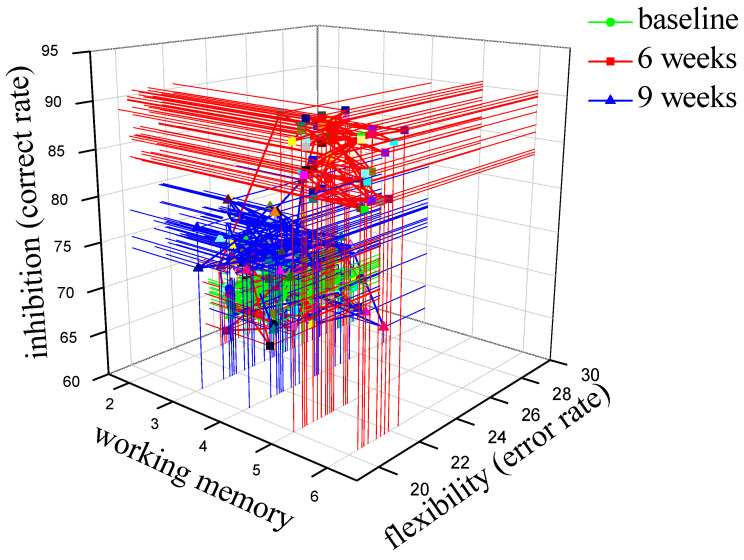
Changes in the three core functions of executive function.

In order to analyze the improvement of the executive function of children with ASD more intuitively, the three main core functions of executive function were analyzed overall (Figure 4). The green lines and points represent the baseline, the red lines and points represent the test results after six weeks, and the blue lines and points represent the test results after nine weeks. We can see that the general trend of the three core functions of executive functions was that they had been enhanced after training, but they had shown a downward trend after the end of training. It can be considered that training would lead to changes in the three core functions of executive function, leading to the enhancement of executive function.

## 4. Discussion

This study investigated the effects of virtual training and physical exercise on the executive function of children with ASD for the duration of six weeks. In the ninth week (three weeks after the intervention), the executive function of children with ASD was also measured. Through the results of three measurements, it was found that virtual training and physical exercise improved the executive function of children with ASD. Once the training was terminated, the executive function of children with ASD had a downward trend; however, it can still maintain a certain effect. The following results will be discussed.

There have been many previous studies trying to prove the impact of exercise intervention on children’s executive function. A meta-analysis of Xue found that the improvement of executive function was related to exercise time [31]. A meta-analysis by Moreau showed that there was no difference of executive function between high-intensity training and medium-intensity training [32]. However, the latest meta-analysis showed that acute and chronic exercise can effectively improve the executive function of adolescents [33]. The inspiration from these studies was that different types of physical exercise may also cause different measurement results on the executive function, which is also a direction we need to study in the future. In a meta-analysis of exercise intervention in children with ASD, Verburgh [34] found that exercise had a better effect on improving executive function in children with ASD, although there were few current studies. On the basis of the existing literature, we conducted a horizontal comparison of the intervention effects of virtual training and physical exercise, and compared the improvement effects of the two different programs on executive function of children. Specifically, after six weeks of virtual training, the executive functions of the virtual training group were improved, including working memory, inhibition, and flexibility. Similarly, after performing physical exercise on the physical exercise group, the executive function of the physical exercise group had also been improved, and the improvement effect was the same as virtual training. However, when the training was stopped, whether it was the virtual training group or the physical exercise group, the executive function of the ASD children would decline, which proved that the executive function brought about by the training was available. Research in recent years has shown that physical exercise can effectively improve people’s cognitive ability [35,36,37], which was consistent with the results in this study that physical exercise can improve the executive function of children with ASD. Through this study, we have concluded that virtual training can also effectively improve children’s executive function, and its effect is consistent with the effect of physical exercise. There was some literature that supports our view, such as studies showing that exercise games can significantly improve the executive function of children with ASD [13,38].

This study showed that virtual training can effectively improve the working memory, inhibition, and flexibility of children with ASD. The reason for this phenomenon may be that the means of virtual training were different, so the results were different. Some studies have proved that virtual training can improve the executive function of children, adolescents and the elderly [39,40,41]. Milajerdi’s research suggested that virtual training can improve the executive function of ASD children better than exercise [39]. However, our study found that virtual training cannot improve the executive function of ASD children more effectively, and virtual training and physical exercise had the same intervention effect on executive function. However, there were still many debates on the improvement of executive function through virtual training [42,43]. One possible reason is that there are many types of games, including immersive games and non-immersive games [44,45]. These different types of games may also lead to different effects on the executive function of children with ASD. Through our study, we also found that after three weeks of stopping the training, the executive function of both virtual training and physical exercise groups decreased, which indicated that the executive function would decrease after stopping training. The possible reasons for this phenomenon are as follows. Brain-derived neurotrophic factor (BDNF) is related to people’s cognitive function. After a certain stimulus, BNDF is synthesized, so cognitive ability is improved [46]. Some studies showed that physical exercise can promote the synthesis of BNDF and enhance the cognitive ability of subjects [47]. However, there was a lack of research on whether virtual training could promote the release of BNDF. We did not measure BNDF, so we merely conjectured that exercise intervention and virtual training would promote BNDF synthesis. This study proved that virtual training can also improve the executive function of children with ASD. It is possible that virtual training has the same effect on BNDF synthesis and physical exercise. However, this study showed that after stopping physical exercise, the executive function of children with ASD was observed to show a downward trend. The same situation occurred in the virtual training group. The explanation for this situation is that, after stopping practice, due to the lack of stimulation, executive function will decline.

The advantage of our study was to compare the executive function of ASD children horizontally through virtual training and physical exercise, and compare the changes of executive function after intervention. However, the current research also has some limitations, which need further discussion. We only perform the test on working memory, inhibition and flexibility of the core of the executive function. Although this reflects the situation of the executive function, it cannot completely evaluate the executive function. Because of the three measurements, we try to not repeat any test; however, we have not discussed whether the influence of practice effects can be completely eliminated. Future research should conduct a comprehensive evaluation of executive function so that we can fully understand the effects of virtual training and physical exercise on the executive function of ASD children.

## 5. Conclusions

The results of this study showed that six weeks of virtual training and physical exercise can improve the executive function of children with ASD. However, after the training ceased, the executive function of children with ASD showed a downward trend. Virtual training and physical exercise improved the executive function of children with ASD in the short term, which reveals that virtual training and physical exercise can be considered to improve the executive function of children with ASD.

## Figures and Tables

**Table 1:** Baseline characteristics of subjects (M ± SD).

Characteristics	Virtual Training Group	Physical Exercise Group	Control Group	*p*-Value
N	34	33	33	—
Age (years)	12.5 ± 2.36	13.1 ± 2.97	12.8 ± 2.69	0.511
Gender (boys/girls)	20/14	17/16	18/15	0.833
Body height (cm)	153.2 ± 10.51	157.8 ± 9.89	155.9 ± 9.73	0.103
Body mass (kg)	52.8 ± 7.74	55.3 ± 6.83	52.3 ± 9.49	0.184
BMI (kg/m^2^)	21.5 ± 2.65	22.4 ± 2.55	21.8 ± 2.76	0.209
CARS	32.3 ± 2.98	32.5 ± 2.65	31.8 ± 3.32	0.201

Abbreviations: M, mean; SD, standard deviation; BMI, body mass index; CARS, Childhood Autism Rating Scale.

## Data Availability

The data presented in this study are available on request from the corresponding author. The data are not publicly available due to privacy reasons.
